# The study of distance changes between lumbar bi-cortical pedicle screws and anterior large vessels in patients with lumbar spondylolisthesis

**DOI:** 10.1186/s12891-021-04811-7

**Published:** 2021-11-01

**Authors:** Li Zhao, Chenguang Wan, Shuhong Han, Baofeng Li, Shaoyi Zheng

**Affiliations:** 1 Department of Cardiovascular surgery, Nanfang Hospital of Southern Medical University, Guangzhou, 510000 Guangdong China; 2 Department of Neurosurgery, Tianjin First Central Hospital, Tianjin, 300000 China; 3 Department of Spine Surgery, Affiliated Hospital of Chengde Medical College, Chengde, 067000 Hebei China; 4 Department of Orthopedics, General Hospital of Southern Theater Command of PLA, The first School of Clinical Medicine, Southern Medical University, Guangzhou, 510000 China

**Keywords:** Lumbar spondylolisthesis, Bi-cortical pedicle screw, Screw location, Lumbar intervertebral disc, Blood vessels

## Abstract

**Objective:**

This paper was a anatomical radiographic study of distance between lumbar bi-cortical pedicle screws (BPSs) and anterior large vessels (ALVs) in patients with lumbar spondylolisthesis, and to provide clinical basis for evaluating the safety of bi-cortical pedicle screw implantation during lumbar spondylolisthesis.

**Methods:**

Complete Computed tomography (CT) data of 104 patients with grade I lumbar spondylolisthesis (L4 52 and L5 52) and 107 non-spondylolisthesis patients (control group) were collected in this study. The distances between lumbar 4,5(L4,5) and sacrum 1(S1) BPSs and ALVs (abdominal aorta, inferior vena cava, left and right common iliac artery, internal and external iliac artery) were respectively measured at different transverse screw angles (TSAs) (L4:5°,10°; L5:10°,15°; S1:0°,5°,10°) and analyzed by SPSS (v25.0). There were three types of distances from the anterior vertebral cortex (AVC) to the ALVs (D_AVC-ALV_): D_AVC-ALV_ N, D_AVC-ALV_ ≥ 0.50 cm, and D_AVC-ALV_ < 0.50 cm; these different distances represented non-contact, distant and close ALV respectively.

**Results:**

We calculated the incidences of screw tip contacting large vessels at different TSAs and provided the appropriate angle of screw implantation. In non-spondylolisthesis group, in L4, the appropriate left TSA was 5°, and the incidence of the close ALV was 4.62%. In S1, the appropriate left TSA was 0° and the incidence of the close ALV was 22.4%, while the appropriate right TSA was 10° and the incidence of the close ALV was 17.8%. In L4 spondylolisthesis group, in L4, the appropriate left TSA was 5°, and the incidence of the close ALV was 3.8%. In L5 spondylolisthesis group, in S1, the appropriate left TSA was 0° and the incidence of the close ALV was 19.2%, while the appropriate right TSA was 10° and the incidence of the close ALV was 21.2%. The use of BPS was not appropriate on the right side of L4 or on the either side of L5 both in spondylolisthesis and control group. In patients with lumbar 4 spondylolisthesis, the incidences of screw tip contacting large vessels were less than the control group in both L4 and 5. In patients with lumbar 5 spondylolisthesis, the incidences of screw tip contacting large vessels were less than the control group in L5, while there were no significant difference in S1.

**Conclusion:**

It is very important that considering the anatomical relationship between the AVC and the ALVs while planning BPSs. The use of BPS does not apply to every lumbar vertebra. In patients with lumbar spondylolisthesis and non-spondylolisthesis patients, the incidences of screw tip contacting large vessels are different.

## Background

Lumbar spondylolisthesis is caused by congenital dysplasia, trauma, strain and other reasons, resulting in the translation of 1 vertebral body over the other and causing instability of the segment [1]. In patients with osteoporosis, due to the poor bone quality, the anti-pullout force of the pedicle screws is significantly inadequate. The screw loosening may lead to operation failure [2].

Traditional methods to improve the stability of pedicle screw are described in detail below:(1) Increased the depth of pedicle screw [3], (2) Using bone cement to strengthen the fixation of pedicle screws [4], (3) Improving the design of pedicle screw, such as expansion screw [5]; First and foremost, Although bone cement augmentation of a pedicle screw is considered a reliable and feasible method up to decreased the incidence of screw loosening, it bears a risk of cement leakage and pulmonary embolism [6]. Secondly, A G Brantley et al. [4] found that screw size had little effect on fixed stiffness in patients with osteoporosis. Third and last, an increase in depth of insertion of the pedicle screw to form a BPS results in higher pullout force and energy [3]. The stress was dispersed between the two cortical bones [7], so that the fixation strength of cortical bone was significantly higher than the cancellous bone. To our knowledge, there were presently little reports on the anatomical structure of ALVs and their association with BPSs.

Therefore, we used imaging methods to study and analyze the lumbar CT images data of patients with lumbar spondylolisthesis and non-spondylolisthesis, and measured the distance between BPSs and ALVs respectively, so as to improve the operation safety and provide anatomical basis.

## Materials and methods

### General information

Inclusion criteria: ① Patients, who underwent lumbar disc CT scan (L3-S1) in the Affiliated Hospital of Chengde Medical University from April 2018 to September 2020, were included in our analysis; ② the imaging data were clear and high-quality, and there was no foreign body artifact;

Exclusion criteria: ① CT image data of non-Affiliated Hospital of Chengde Medical University, with unclear display; ② patients diagnosed with scoliosis, vertebral fracture, tumor, tuberculosis or transversal vertebrae; ③ patients with lumbar or retroperitoneal surgery history affecting normal anatomy.

## Method

The Meyerding classification grade [8] is determined by measuring the degree of slip. The classification system divides slip into five grades: 0 to 25% is Grade I, 25 to 50% is Grade II, 50 to 75% is Grade III, 75 to 100% is Grade IV, and greater than 100% is Grade V. This article selects patients with grade I slippage in L4 and L5.

**Fig. 1:**
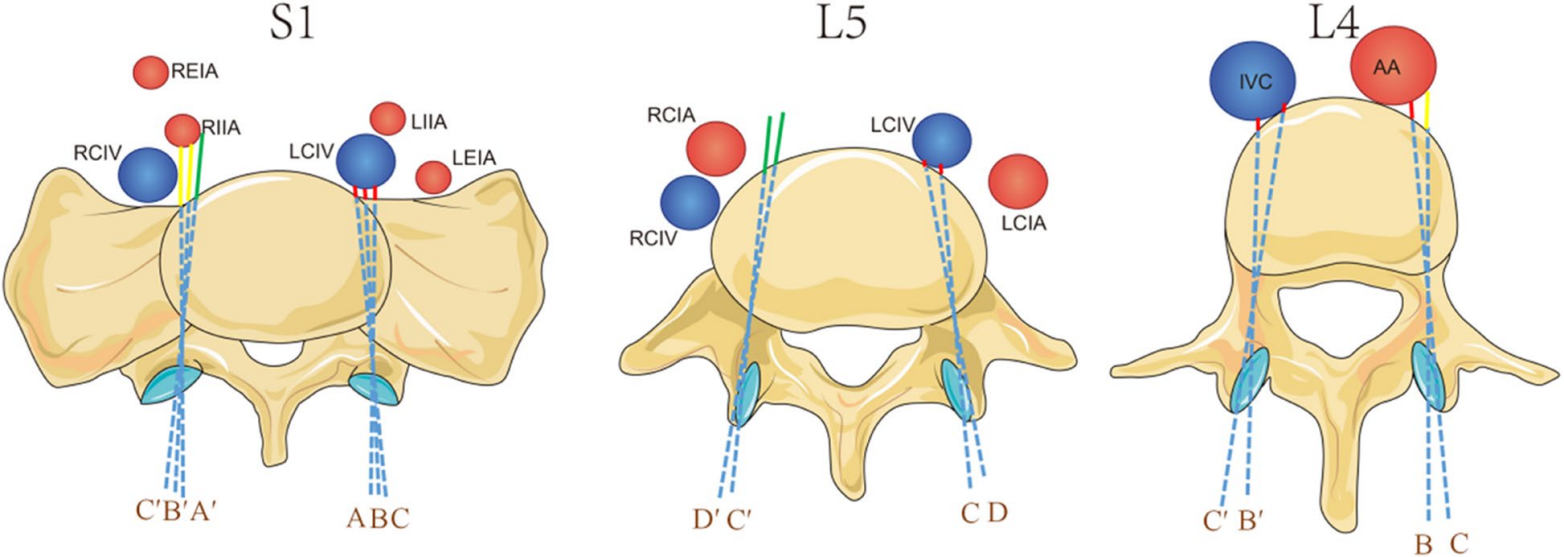
The measurement of distance between lumbar bi-cortical pedicle screws and anterior large vessels in L4, L5, S1 (AA: abdominal aorta, IVC: inferior vena cava, LCIA: left common iliac artery, RCIA: right common iliac artery, LCIV: left common iliac vein, RCIV: right common iliac vein, LEIA: left external iliac artery, LIIA: left internal iliac artery, REIA: right external iliac artery, RIIA: right internal iliac artery. A, B, C, D respectively represent the left TSA of the BPS at 0°, 5°, 10° and 15°; A’, B′, C′ and D′ respectively represent the right TSA of the BPS at 0°, 5°, 10°, and 15°)

Xinru Du et al. [9, 10] found that when the L4 TSA is 5–10°, the L5 TSA is 10–15°and the S1 TSA is 0–10°, the pedicle screw passes through the central axis of the pedicle, which is the connecting line between the screw placement point and the midpoint of the pedicle stenosis at different TSAs. According to the author’s suggestion, we assumed the pedicle screw is on the central axis of the pedicle and respectively measured the distances between BPSs in L4,5 and S1 and ALVs at different TSAs (L4:5°,10°; L5:10°,15°; S1:0°,5°,10°) (Fig. 1). According to the D_AVC-ALV_ different [11], the distance between AVC and ALV was classified into three types: non-contact, distant and close ALVs. If the prolonged line segment of the BPS did not contact the ALV, it was indicated as “D_AVC-ALV_ N” which meant a non-contact ALV. If the prolonged line segment of the BPS contacted the ALV and the distance was larger or equal to 0.50 cm, it was indicated as “D_AVC-ALV_ ≥0.50 cm” which meant a distant ALV. If the prolonged line segment of the BPS contacted the ALV and the distance was less than 0.50 cm, it was expressed as “D_AVC-ALV_ < 0.50 cm” which meant a close ALV. Then, we calculated the incidences of these three types of ALV at each TSA of the BPSs in L4, L5 and S1. The higher the incidence of the close ALV, the higher the risk of injury potential to the ALV. We also collected the participants’ age and sex.

### Statistical data processing

The measurement data were expressed as mean ± standard deviation and t-test was adopted. We use χ2 test to test the counting data. With SPSS (V25.0) for statistical analysis, when the *p*-value less than 0.05 (*p* < 0.05), the difference was statistically significant.

## Results

### General information of research objects

In this study,104 cases of lumbar spondylolisthesis (L4 52 cases, L5 52 cases), including 27 males and 77 females. There were 107 cases in the control group, including 38 males and 69 females. There was no significant difference in gender composition between the two groups (χ^2^ = 2.258, *p* = 0.133 > 0.05). (Table 1).

**Table 1:** sex composition and age distribution of subjects

Group	Total	Male	Female	Age (years)
Spondylolisthesis group	104	27	77	60.13 ± 11.305
Control group	107	38	69	30.32 ± 9.031^①^

*Note*: ① compared with spondylolisthesis group, *P* < 0.05

### Comparison of the distance between BPSs and ALVs in both the control group and **spondylolisthesis group**

In the control group, in L4, the incidences of D_AVC-ALV_ N, D_AVC-ALV_ ≥ 0.50 cm, and D_AVC-ALV_ < 0.50 cm between the left and right sides were significant difference at 5° and 10° respectively (*p* < 0.05). The lowest incidence of the close ALVs on the left side of L4 was 9.3% at 5°, while the right side was 73.8% at 10°. In L5, the incidences of D_AVC-ALV_ N, D_AVC-ALV_ ≥ 0.50 cm, and D_AVC-ALV_ < 0.50 cm between the left and right sides were not significant difference at and 10° and 15° respectively (*p* > 0.05). The lowest incidence of the close ALVs on the left side of L5 was 75.7% at 10°, while the right side was 73.8% at 10°. In S1, the incidences of D_AVC-ALV_ N, D_AVC-ALV_ ≥ 0.50 cm, and D_AVC-ALV_ < 0.50 cm between the left and right sides were significant difference at 5° and 10° respectively (*p* < 0.05). But there were not significant difference at 0 ° (*p* > 0.05). The lowest incidence of the close ALVs on the left side of S1 was 22.4% at 0°, while the right side was 17.8% at 10°.

In the L4 spondylolisthesis group, in L4, the incidences of D_AVC-ALV_ N, D_AVC-ALV_ ≥ 0.50 cm, and D_AVC-ALV_ < 0.50 cm between the left and right sides were significant difference at 5° and 10° respectively (*p* < 0.05). The lowest incidence of the close ALVs on the left side of L4 was 3.8% at 5°, while the right side was 36.5% at 10°. In L5, the incidences of D_AVC-ALV_ N, D_AVC-ALV_ ≥ 0.50 cm, and D_AVC-ALV_ < 0.50 cm between the left and right sides were not significant difference at 10° and 15° respectively (*p* > 0.05). The lowest incidence of the close ALVs on the left side of L5 was 53.8% at 10°, while the right side was 46.2% at 10°.

In the L5 spondylolisthesis group, in L5, the incidences of D_AVC-ALV_ N, D_AVC-ALV_ ≥ 0.50 cm, and D_AVC-ALV_ < 0.50 cm between the left and right sides were not significant difference at 10° and 15° respectively (*p* > 0.05). The lowest incidence of the close ALVs on the left side of L5 was 50% at 10°, while the right side was 51.9% at 15°. In S1, the incidences of D_AVC-ALV_ N, D_AVC-ALV_ ≥ 0.50 cm, and D_AVC-ALV_ < 0.50 cm between the left and right sides were significant difference at 5° and 10° respectively (*p* < 0.05). But there were not significant difference at 0 ° (*p* > 0.05). The lowest incidence of the close ALVs on the left side of S1 was 19.2% at 0°, while the right side was 21.2% at 0° and 10°. (Table 2).

**Table 2:** Comparison of the distance between BPSs and ALVs in both the control group and spondylolisthesis group

Lumbar	TSA	Type	Left Number (%)	Right Number (%)	P_1_	Left D_AVC-ALV _(cm)	Right D_AVC-ALV_ (cm)	P_2_
Control group								
L4	5	N	58 (54.2%)	2 (1.9%)				
		≥	39 (36.4%)	14 (13.1%)	0.000	0.99 ± 0.13	0.57 ± 0.03	0.000
		<	10 (9.3%)	91 (85%)		0.33 ± 0.07	0.28 ± 0.07	0.013
L4	10	N	16 (15%)	4 (3.7%)				
		≥	60 (56.1%)	24 (22.4%)	0.000	0.78 ± 0.08	0.66 ± 0.07	0.000
		<	31 (29%)	79 (73.8%)		0.31 ± 0.04	0.16 ± 0.06	0.000
L5	10	N	8 (7.5%)	11 (10.3%)				
		≥	18 (16.8%)	17 (15.9%)	0.768	0.7 ± 0.05	0.58 ± 0.03	0.000
		<	81 (75.7%)	79 (73.8%)		0.11 ± 0.04	0.07 ± 0.06	0.000
L5	15	N	5 (4.7%)	15 (14%)				
		≥	4 (3.7%)	4 (3.7%)	0.063	0.68 ± 0.03	0.61 ± 0.029	0.073
		<	98 (91.6%)	88 (82.2%)		0.12 ± 0.05	0.12 ± 0.05	0.749
S1	0	N	21 (19.6%)	20 (18.7%)				
		≥	62 (57.9%)	65 (60.7%)	0.913	0.95 ± 0.22	0.75 ± 0.07	0.000
		<	24 (22.4%)	22 (20.6%)		0.21 ± 0.1	0.31 ± 0.07	0.000
S1	5	N	18 (16.8%)	29 (27.1%)				
		≥	11 (10.3%)	46 (43%)	0.000	1.04 ± 0.1	0.79 ± 0.11	0.000
		<	78 (72.9%)	32 (29.9%)		0.26 ± 0.08	0.24 ± 0.06	0.353
S1	10	N	40 (37.4%)	87 (81.3%)				
		≥	11 (10.3%)	1 (0.9%)	0.000	1.03 ± 0.09	0.5	0.000
		<	56 (52.3)	19 (17.8%)		0.18 ± 0.09	0.39 ± 0.04	0.000
L4 spondylolisthesis group								
L4	5	N	42 (80.8%)	12 (23.1%)				
		≥	8 (15.4%)	19 (36.5%)	0.000	1.24 ± 0.05	0.73 ± 0.06	0.000
		<	2 (3.8%)	21 (40.4%)		0.39 ± 0.03	0.42 ± 0.04	0.365
L4	10	N	17 (32.7%)	8 (15.4%)				
		≥	27 (51.9%)	19 (36.5%)	0.001	0.88 ± 0.1	0.78 ± 0.06	0.000
		<	8 (15.4%)	25 (48.1%)		0.43 ± 0.04	0.39 ± 0.04	0.021
L5	10	N	8 (15.4%)	12 (23.1%)				
		≥	16 (30.8%)	16 (30.8%)	0.575	0.76 ± 0.05	0.75 ± 0.07	0.742
		<	28 (53.8%)	24 (46.2%)		0.2 ± 0.05	0.15 ± 0.05	0.001
L5	15	N	9 (17.3%)	15 (28.8%)				
		≥	8 (15.4%)	8 (15.4%)	0.357	0.8 ± 0.05	0.7 ± 0.05	0.012
		<	35 (67.3%)	29 (55.8%)		0.22 ± 0.07	0.19 ± 0.06	0.043
L5 spondylolisthesis group								
L5	10	N	10 (19.2%)	8 (15.4%)				
		≥	16 (30.8%)	13 (25%)	0.54	0.79 ± 0.07	0.71 ± 0.06	0.001
		<	26 (50%)	31 (59.6%)		0.24 ± 0.05	0.17 ± 0.05	0.000
L5	15	N	9 (17.3%)	17 (32.7%)				
		≥	9 (17.3%)	8 (15.4%)	0.088	0.83 ± 0.08	0.78 ± 0.07	0.259
		<	34 (65.4%)	27 (51.9%)		0.26 ± 0.08	0.23 ± 0.06	0.1
S1	0	N	11 (21.2%)	11 (21.2%)				
		≥	31 (59.6%)	30 (57.7%)	0.969	0.99 ± 0.07	0.7 ± 0.04	0.000
		<	10 (19.2%)	11 (21.2%)		0.2 ± 0.23	0.29 ± 0.04	0.000
S1	5	N	10 (19.2%)	14 (26.9%)				
		≥	6 (11.5%)	24 (46.2%)	0.000	0.91 ± 0.03	0.82 ± 0.06	0.001
		<	36 (69.2%)	14 (26.9%)		0.21 ± 0.07	0.2 ± 0.06	0.784
S1	10	N	21 (40.4%)	41 (78.8%)				
		≥	5 (9.6%)	0	0.000	1 ± 0.05		
		<	26 (50%)	11 (21.2%)		0.13 ± 0.07	0.41 ± 0.04	0.000

*Note*: N, ≥ and < represented non-contact, distant and close ALV respectively

### Effect of lumbar spondylolisthesis on the distance between BPSs and ALVs in L4, L5 and S1

In the L4 spondylolisthesis group, in L4, the incidences of D_AVC-ALV_ N, D_AVC-ALV_ ≥ 0.50 cm, and D_AVC-ALV_ < 0.50 cm between the spondylolisthesis and control group were significant difference at 5° and 10° respectively (*p* < 0.05). In L5, the incidences of D_AVC-ALV_ N, D_AVC-ALV_ ≥ 0.50 cm, and D_AVC-ALV_ < 0.50 cm between the spondylolisthesis and control group were significant difference at 10° and 15° respectively (*p* < 0.05) (Table 3).

**Table 3:** Effect of lumbar 4 spondylolisthesis on the distance between BPSs and ALVs

Group		L4						L5					
		5			10			10			15		
		*N*	≥	<	*N*	≥	<	*N*	≥	<	*N*	≥	<
Left	C	58 (54.2%)	39 (36.4%)	10 (9.3%)	16 (15%)	60 (56.1%)	31 (29%)	8 (7.5%)	18 (16.8%)	81 (75.7%)	5 (4.7%)	4 (3.7%)	98 (91.6%)
	S	42 (80.8%)	8 (15.4%)	2 (3.8%)	17 (32.7%)	27 (51.9%)	8 (15.4%)	8 (15.4%)	16 (30.8%)	28 (53.8%)	9 (17.3%)	8 (15.4%)	35 (67.3%)
	P		0.004			0.018			0.02			0.001	
Right	C	2 (1.9%)	14 (13.1%)	91 (85%)	4 (3.7%)	24 (22.4%)	79 (73.8%)	11 (10.3%)	17 (15.9%)	79 (73.8%)	15 (14%)	4 (3.7%)	88 (82.2%)
	S	12 (23.1%)	19 (36.5%)	21 (40.4%)	8 (15.4%)	19 (36.5%)	25 (48.1%)	12 (23.1%)	16 (30.8%)	24 (46.2%)	15 (28.8%)	8 (15.4%)	29 (55.8%)
	P		0.000			0.000			0.003			0.001	

*Note*: *C* Control group, *S* Spondylolisthesis group, *N*, ≥ and < represented non-contact, distant and close ALV respectively

In the L5 spondylolisthesis group, in L5, the incidences of D_AVC-ALV_ N, D_AVC-ALV_ ≥ 0.50 cm, and D_AVC-ALV_ < 0.50 cm between the spondylolisthesis and control group were significant difference at 10° and 15° respectively (*p* < 0.05). In S1, the incidences of D_AVC-ALV_ N, D_AVC-ALV_ ≥ 0.50 cm, and D_AVC-ALV_ < 0.50 cm between the spondylolisthesis and control group were not significant difference at 0°, 10° and 15° respectively (*p* > 0.05) (Table 4).

**Table 4:** Effect of lumbar 5 spondylolisthesis on the distance between BPSs and ALVs

Group		L5						S1								
		10			15			0			5			10		
		*N*	≥	<	*N*	≥	<	*N*	≥	<	*N*	≥	<	*N*	≥	<
Left	C	8 (7.5%)	18 (16.8%)	81 (75.7%)	5 (4.7%)	4 (3.7%)	98 (91.6%)	21 (19.6%)	62 (57.9%)	24 (22.4%)	18 (16.8%)	11 (10.3%)	78 (72.9%)	40 (37.4%)	11 (10.3%)	56 (52.3%)
	S	10 (19.2%)	16 (30.8%)	26 (50%)	9 (17.3%)	9 (17.3%)	34 (65.4%)	11 (21.2%)	31 (59.6%)	10 (19.2%)	10 (19.2%)	6 (11.5%)	36 (69.2%)	21 (40.4%)	5 (9.6%)	26 (50%)
	P		0.004			0.000			0.894			0.89			0.935	
Right	C	11 (10.3%)	17 (15.9%)	79 (73.8%)	15 (14%)	4 (3.7%)	88 (82.2%)	20 (18.7%)	65 (60.7%)	22 (20.6%)	29 (27.1%)	46 (43%)	32 (29.9%)	87 (81.3%)	1 (0.9%)	19 (17.8%)
	S	12 (23.1%)	16 (30.8%)	24 (46.2%)	17 (32.7%)	8 (15.4%)	27 (51.9%)	11 (21.2%)	30 (57.7%)	11 (21.2%)	14 (26.9%)	24 (46.2%)	14 (26.9%)	41 (78.8%)	0	11 (21.2%)
	P		0.003			0.000			0.919			0.91			0.777	

*Note*: *C* Control group, *S* Spondylolisthesis group; *N*, ≥ and < represented non-contact, distant and close ALV respectively

Based on the above results, we found that in patients with lumbar 4 spondylolisthesis, the incidences of screw tip contacting large vessels were less than the control group in both L4 and 5, and in patients with lumbar 5 spondylolisthesis, the incidences of screw tip contacting large vessels were less than the control group in L5, while there were no significant difference in S1.

## Discussion

The BPS insertion method was initially used for S1 [12]. In this text, the authors clarify the safety scope of BPSs. In recent years, there are numerous clinical studies that have confirmed the feasibility and mechanical advantages of BPSs [13–15]. In 2015, Le Cann et al. [7] through using pig lumbar spine concluded that the pedicle screw fixation device needs to be bi-cortical to enhance stability in young animals. In the same year, Karami et al. [3] through osteoporotic cadaveric lumbar spines concluded that additional purchase of the stiff anterior cortex is indispensable for achieving superior screw-bone structure stability and rigidity. The advantages of BPS fixation were confirmed by animal experiments and cadaveric experiments. Thus, we measured the distance between BPSs and ALVs in order to reduce the incidence of vascular injury.

Shinya Okuda et al. [16, 17] reported that complications associated with pedicle screws misplacement. The intraoperative Complications they reported were dural tearing, pedicle screw malposition and nerve injury. The early postoperative complications they reported were pulmonary, cardiac, and cerebrovascular morbidity, hardware failure, infection and neurological complications. The late postoperative complications they reported were hardware failure, nonunion, late infection, and adjacent-segment degeneration. To improve the accuracy of pedicle screw placement and to reduce the complications associated with pedicle screws misplacement, we can use imaging-guided navigation.

Imaging-guided navigation can provide the surgeon with additional anatomical information to increase precision of setting pilot holes for pedicle screws [18]. In a meta-analysis for placement of pedicle screws in the spine, image-guided navigation showed a higher accuracy 95.5% compared with 91.5% for freehand placement [19]. Previous studies have summarized several approaches of image-guided navigation, which included fluoroscopy-assisted, computed tomography image navigation, and robot-assisted [20]. In 2019, through retrospective analysis of the 51,161 pedicle screw cases, Alexander Perdomo-Pantoja et al. [21] concluded that the CT provides the highest pedicle screw placement accuracy and lowest rates of revision, compared with other techniques of image-guided navigation.

Placing BPSs precisely is essential to bi-cortical fixation, as protruding screw tips can damage ALVs [13, 22]. In order to place BPSs precisely, the depth and angle of screw must be planned before operation. Previous studies [23, 24] indicate that the safe distance rage of the BPSs protruding tips should be maintained within the 5 mm. This is essential for the secure use of BPSs to improve the strength of fixation. Our finding suggests that in non-spondylolisthesis group, the appropriate left TSA was 5° in L4, the appropriate left TSA was 0° in S1, and the appropriate right TSA was 10° in S1. In patients with lumbar 4 spondylolisthesis, the appropriate left TSA was 5° in L4. In patients with lumbar 5 spondylolisthesis, the appropriate left TSA was 0° in S1 and the appropriate right TSA was 10° in S1. However, The BPSs insertion method were not suitable for every lumbar spine. The use of BPS was not appropriate on the right side of L4 or on the either side of L5, which is similar with the finding result of Liehua Liu et al. [11]. In addition, the author also concluded the recommended TSA of each BPS of L1-L3. Meanwhile, we also find that in patients with lumbar 4 spondylolisthesis, the incidences of screw tip contacting large vessels were less than the control group in both L4 and 5 and in patients with lumbar 5 spondylolisthesis, the incidences of screw tip contacting large vessels were less than the control group in L5, while there were no significant difference in S1. The reasons are as follows: Patients with lumbar spondylolisthesis often occur labial hyperosteogeny on the upper and lower margin of the vertebral body, and the hyperplastic osteophytes push the anterior longitudinal ligament and large vessels forward, thus increasing the distance between BPSs and ALVs [25], while, at the level of L5-S1 intervertebral disc, the abdominal aorta and inferior vena cava have bifurcated into the left and right common iliac arteries and veins, and some patients have even bifurcated into internal and external iliac arteries and veins, and for these patients, the operative window of intervertebral disc surgery has a large range [26], and the hyperplastic osteophytes may avoid the large vessels. Thus, we think that in S1, there were no significant difference in the incidences of large vessels between spondylolisthesis and non-spondylolisthesis group.

Luis Marchi et al. [22] summarize different factors which may affect the closest distance between the lumbar spine and anterior large vessels. One of them is lumbar lordosis. The author’s results show that lordosis did not significantly affect the closest distance between the lumbar spine and anterior large vessels at any level. The reasons may be as follows: when the lumbar lordosis increases, the abdominal aorta and inferior vena cava will also produce physiological lordosis, but the relative position with the lumbar vertebral body remains unchanged.

In order to avoid damage to the ALVs, we suggest that each surgeon must make his own surgical strategy, with due consideration of preoperative imaging examination. First and foremost, the BPS insertion method suggested in this research can not completely prevent damage to the ALV. Secondly, if necessary, patient should undergo a computed tomography angiography (CTA) examination before operation, which can be used to identify the angle and depth of the BPS. Third and last, it can be considered safe when the protruding tip of the pedicle screws to be less than 3 mm [23].

There are several limitations in our study. Firstly, there are inevitable measurement in the experimental data, and the clinical effect not studied. Secondly, The CT images used as a reference for placing BPSs are taken commonly when the patient is supine position, while the traditional operative position is prone. In order to the effect of body positions on the distance between BPSs and ALVs, Riccio A et al. [27] through experiments found that the inferior vena cava and the abdominal aorta in the lumbar region is relatively immobile in the prone and supine positions. Thus, we believe that the distance between BPSs and ALVs has no significant difference in different body positions. Future studies can investigate the relative distance between BPSs and the ALVs in different body positions.

To sum up, the use of BPS does not apply to every lumbar vertebra. When placing BPSs, we present the appropriate TSAs in L4 and S1. In patients with lumbar spondylolisthesis and non-spondylolisthesis patients, the incidences of screw tip contacting large vessels are different.

## Data Availability

The datasets used and/or analyzed during the current study are available from the corresponding author on reasonable request. Readers can access the data and material supporting the conclusions of the study by contacting Li Zhao at 654720450@qq.com.

## Abbreviations

BPSs: Bi-cortical pedicle screws
ALVs: Anterior large vessels
CT: Computed tomography
AVC: Anterior vertebral cortex
CTA: Computed tomography angiography
